# Emergency Hospital Visits in Association with Volcanic Ash, Dust Storms and Other Sources of Ambient Particles: A Time-Series Study in Reykjavík, Iceland 

**DOI:** 10.3390/ijerph120404047

**Published:** 2015-04-13

**Authors:** Hanne Krage Carlsen, Thorarinn Gislason, Bertil Forsberg, Kadri Meister, Throstur Thorsteinsson, Thorsteinn Jóhannsson, Ragnhildur Finnbjornsdottir, Anna Oudin

**Affiliations:** 1 Centre of Public Health Sciences, University of Iceland, Stapi v/Hringbraut, 101 Reykjavík, Iceland; E-Mail: rgf1@hi.is; 2 Unit of Occupational and Environmental Medicine, Department of Public Health and Clinical Medicine, Umeå University, 90187 Umeå, Sweden; E-Mails: bertil.forsberg@envmed.umu.se (B.F.); kadri.meister@envmed.umu.se (K.M.); anna.oudin@envmed.umu.se (A.O.); 3 Department of Respiratory Medicine and Sleep, Landspítali University Hospital-Fossvogur, 108 Reykjavík, Iceland; 4 Faculty of Medicine, School of Health Sciences, University of Iceland, Vatnsmýrarvegi 16, 101 Reykjavík, Iceland; E-Mail: thorarig@landspitali.is; 5 Unit of Environment and Natural Resources, University of Iceland, Sturlugata 7, 101 Reykjavík, Iceland; 6 Institute of Earth Sciences, School of Engineering and Natural Sciences, University of Iceland, Sturlugata 7, 101 Reykjavík, Iceland; E-Mail: throsturth@hi.is; 7 Environmental Agency of Iceland, Suðurlandsbraut 24, 108 Reykjavík, Iceland; E-Mail: thorsteinnj@umhverfisstofnun.is

**Keywords:** particle matter, volcanic ash, dust storms, emergency hospital visits, respiratory health, cardiovascular health

## Abstract

Volcanic ash contributed significantly to particulate matter (PM) in Iceland following the eruptions in Eyjafjallajökull 2010 and Grímsvötn 2011. This study aimed to investigate the association between different PM sources and emergency hospital visits for cardiorespiratory causes from 2007 to 2012. Indicators of PM_10_ sources; “volcanic ash”, “dust storms”, or “other sources” (traffic, fireworks, and re-suspension) on days when PM_10_ exceeded the daily air quality guideline value of 50 µg/m^3^ were entered into generalized additive models, adjusted for weather, time trend and co-pollutants. The average number of daily emergency hospital visits was 10.5. PM_10_ exceeded the air quality guideline value 115 out of 2191 days; 20 days due to volcanic ash, 14 due to dust storms (two days had both dust storm and ash contribution) and 83 due to other sources. High PM_10_ levels from volcanic ash tended to be significantly associated with the emergency hospital visits; estimates ranged from 4.8% (95% Confidence Interval (CI): 0.6, 9.2%) per day of exposure in unadjusted models to 7.3% (95% CI: −0.4, 15.5%) in adjusted models. Dust storms were not consistently associated with daily emergency hospital visits and other sources tended to show a negative association. We found some evidence indicating that volcanic ash particles were more harmful than particles from other sources, but the results were inconclusive and should be interpreted with caution.

## 1. Introduction

Particle matter (PM) is harmful to cardio-respiratory health [1], but few studies have addressed the importance of the source. As both physical and chemical composition of particle matter (PM) vary between sources it seems plausible that particles from different sources could have different toxicological effects [2,3,4]. PM divides into PM_10,_ coarser particles with an aerodynamic diameter less than 10 µm and fine PM_2.5_ (aerodynamic diameter less than 2.5 µm). Though many studies have found health risks associated with short-term exposure to PM_2.5_ [5], several studies have in recent years found health effects for coarse particles [6,7,8]. Most studies in air pollution epidemiology have been conducted in urban areas, where traffic is the main source, but particles from natural sources have come into focus [9]. 

Dust storms of natural origin are common in Iceland and occur as particles from eroded areas are resuspended [10,11,12].

Resuspended ash from the recent eruptions of Eyjafjallajökull and Grímsvötnwas added to the PM mixture in the capital region in Iceland where several severe volcanic ash events occurred in the years 2010 and 2011 [13,14]. Exposure to recently erupted Eyjafjallajökull ash was associated with elevated prevalence of respiratory irritation symptoms and cough in the immediately affected area [15]. However, little is known about the health effects from exposure to long-range transported volcanic ash. Increased exacerbations of respiratory diseases are frequently reported [16,17,18,19] and possible association with mortality has been suggested for long-transported volcanic ash [20]. Natural dust particles, for example from deserts, have been associated with adverse health outcomes such worsened lung function in asthmatics [21] and mortality [22,23,24]. 

In previous studies from Iceland PM_10_ was associated with dispensing of anti-asthma medication [25], but not with emergency hospital visits for cardiorespiratory causes [26] or dispensing of medication for angina pectoris [27]. However, these studies did not account for source specific effects of this unique mixture of particles. Possible health effects of volcanic ash exposure in the capital area of Iceland are unknown.

This study aims to investigate if indicators different sources of PM_10_; volcanic ash, dust storms, or other local or urban sources (traffic, local resuspension or fireworks) were associated with the daily number of emergency hospital visits due to cardiorespiratory causes in Reykjavik, Iceland from 2007–2012.

## 2. Experimental Section

### 2.1. Health Outcomes

The Landspítali University Hospital is the only tertiary care center in Iceland, located in the capital Reykjavík (population around 200,000). The patient database is based on the international classification of diseases (ICD10) since 2001, and the data are registered and stored using the personal identification number, which is assigned to all permanent residents of Iceland. We extracted data on non-elective emergency room visits and hospital admissions (from now on *emergency hospital visits*) of adults (18 or older) who were residents of the capital area (postcodes 101–225 and postcode 270) for cardiac (ICD codes I20-I27, I46, I48; I50), respiratory (ICD codes J20-J22, J40-J46, J96), and stroke diagnoses (ICD codes I60-I69, G46-G46), from 2007 to 2012 along with a postcode of the patient’s residence, gender, and age at admission. The personal identification number was removed and replaced with a random number that was untraceable but unique to each individual so all his or hers admissions could be kept apart so that each person occurred only once per day in the time series. We obtained the time of influenza epidemics from the directorate of health [28,29].

### 2.2. Pollution Data

The pollution data were obtained from the City of Reykjavík Environmental Branch and the Environmental Agency of Iceland. We used hourly data on PM_10_, NO_2_, H_2_S, temperature, and relative humidity from a single measuring station in urban Reykjavík to calculate daily averages for all the variables from the data. Unfortunately the O_3_ measurements for the latter part of the study period were marred with errors, and therefore O_3_ were excluded from the analysis. 

On days where the air quality guideline value of PM_10_ (50 µg/m^3^) [30] was exceeded, the particle sources were estimated using several steps. Firstly, through direct observations on the day of exceedance by monitoring staff. High values at several urban stations, including urban background, indicated that the source was not local. If correlation was high between PM_10_ and NO_x_ values, traffic was a likely source [31]. The source areas for dust storms are fairly well known [10,12,32,33], as are the new source areas following the 2010 and 2011 eruptions [13,14].Wind speed and wind direction were critical for distinguishing dust and ash storms from local events and sea spray as well as identifying source areas of the event. Trajectories were used for backtracking particle paths to source areas. As dust and ash storms occur during dry conditions, they could often be confirmed using satellite images.

The sources were coded as indicators for *volcanic ash, dust storm*, or *other* (traffic, local resuspension, fireworks, or sources outside Iceland) [10,14,34]. The two days that had contributions from both volcanic ash and dust storm were counted in both categories.

### 2.3. Statistical Methods

Descriptive statistics were calculated for the daily 24-hour mean of pollution data and for the characteristics of the study population. We also calculated the correlation coefficient (Pearson’s R^2^) between the pollution and weather variables. Source specific associations of high levels of PM_10_ were investigated separately for each source indicator, first unadjusted, then adjusting for several combinations of co- pollutants; PM_10_, the gaseous pollutants; NO_2_, and H_2_S, and finally all available pollutants; PM_10_, NO_2_ and H_2_S. All analyses were performed using a generalized additive regression model (GAM) and were adjusted for influenza season with a binary indicator. A cubical cyclical spline of temperature was used to model seasonal associations. Degrees of freedom for the time trend splines were selected to optimize Akaike’s Information Criterion (AIC) in the basic multi-pollutant model without indicators [35]. Degrees of freedom between 3 and 70 were tested. The best value was then used in all models. The time trend spline for the whole period was set at 30 degrees of freedom. Sensitivity analyses were performed on all lags of the exposure variables in order to determine which lags to take into account (see Supplementary Table S1). Lags were selected based on optimizing the model diagnostics (% deviance explained and R^2^) and previous studies [16,36,37]. The models were inspected for auto-correlation.

### 2.4. Reporting

The results were reported as the percent (%) change in number of emergency hospital visits with a 95% confidence interval (CI) associated with one day with high PM_10_ in the previous 7 days (indicator analysis). The R package “mgcv” [38] was used for all statistical analysis. The Data Protection Agency (2010121176AT/), the Bioethics Committee (VSNb2010120017/03.7), and the Hospital ethics board (Letter: 2010/12/22) approved the use of the data.

## 3. Results and Discussion

### 3.1. Results

#### 3.1.1. Descriptive Results

**Figure 1:**
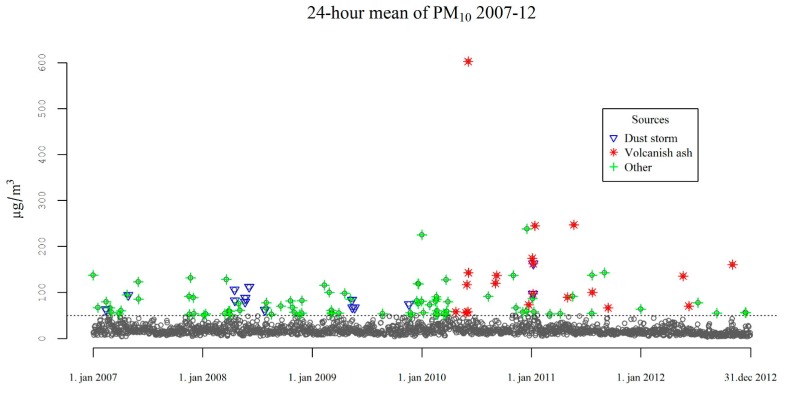
Daily (24-h) averages values of PM_10_ and its sources during exceedance of the air quality guideline value of 50 µg/m^3^ (dotted line).

In total, there were 22,892 emergency hospital visits for cardiorespiratory causes between 2007 and 2012 by a total of 11,567 (54% men) adult individuals. The mean age at first emergency hospital visit within the study period was 68 years (Table 1). There were 115 days in the time series where air quality guideline value of 50 μg/m^3^ was exceeded. The source was (wholly or partially) volcanic ash on 20 days, dust storms on 14 days, and other sources (traffic, resuspension or fireworks) during 83 days (5 days due to fireworks the rest due to traffic and local resuspension) (Table 1, Figure 1). 

**Table 1:** Descriptive statistics of the data and daily emergency hospital visits and daily pollutants during the study period and stratified by time-strata used in the analysis.

Emergency Hospital Visits			2007–2012					
Number of admissions and ER visits			22,892					
Individuals (n)			11,567					
Age at first admission, mean (sd)			68.1 (16.1)					
Percentage female (n)			46.3 (5351)					
**Daily Pollutant Levels**	**2007–2012** **n = 2192**		**Volcanic Ash** **n = 20**		**Dust Storm** **n = 14**		**Other Sources** **n = 83**	
	**Mean**	**sd**	**Mean**	**sd**	**Mean**	**sd**	**Mean**	**sd**
PM_10_ (µg/m^3^)	21.8	23.9	145.5	121.5	88.5	26.3	78.8	35.0
H_2_S (µg/m^3^)	3.2	5.7	3.4	3.4	2.5	5.0	4.0	8.3
NO_2_ (µg/m^3^)	18.2	13.2	8.3	8.1	10.5	8.1	25.2	19.5
Temperature (°C)	6.0	5.3	6.4	7.1	6.9	6.4	5.7	6.4
Relative humidity (%)	76.6	11.0	66.3	7.0	65.7	5.3	67.4	10.6

sd: standard deviation.

Daily mean PM_10_ levels were not higher after the eruption, but were highest during volcanic ash days. NO_2_ levels were highest when high PM_10_ occurred due to other sources than dust storm t and volcanic ash (Table 1). PM_10_ levels were correlated with temperature and relative humidity, whereas NO_2_ and H_2_S were significantly inter-correlated (data not shown).

#### 3.1.2. Results of the Analysis

When the three types of specific sources were combined into one category, there was no association between days with high PM_10_ levels (>50 μg/m^3^) and the number of daily hospital encounters; the effect estimate was −0.7% (95% CI −2.6, 1.4%, *p* = 0.52) unadjusted and −2.8% (95% CI −6.6, 1.2%, *p* = 0.16) fully adjusted (Table 2). High levels of PM_10_ due to volcanic ash were associated with statistically significant increases in emergency hospital visits in the following 7 days; the effect estimates ranged from 4.8% (95% CI 0.6, 9.2%, *p* = 0.02) in the unadjusted model to 7.3% (95% CI −0.4, 15.5%, *p* = 0.06) in the fully adjusted model (Table 2). High levels of PM_10_ due to dust storms were associated with statistically significant increases in emergency hospital visits in unadjusted models and models adjusted for PM_10_; the effect estimates were 5.1% (95% CI 0.5, 9.9%, *p* = 0.03) and 5.8% (95% CI 0.9, 19.9%, *p* = 0.02) respectively. After adjusting for gaseous pollutants there were no statistically significant associations between high levels of PM_10_ due to dust storms and emergency hospital visits, with effect estimates at 2.1% (95% CI −3.3, 8.0%, *p* = 0.44) when adjusting for NO_2_ and H_2_S, and 1.2% (95% CI −4.6, 7.4%, *p* = 0.69) when adjusting for PM_10_, NO_2_, and H_2_S (Table 2). High PM_10_ levels due to other sources (traffic, resuspension, or fireworks) were negatively associated with the daily number of emergency hospital visits by −3.3% (95% CI −5.6, −1.0%, *p* = 0.00) before adjusting for other pollutants, −4.4% (95% CI −7.1, −1.7%, *p* = 0.00) after adjusting for PM_10_, and -3.6% (95% CI −6.9, −0.2%, *p =* 0.04) in the multi-pollutant model. Adjusting for gaseous pollutants, but not for PM_10_ the effect estimate was −1.2% (95% CI −3.8, 1.5%, *p* = 0.38). In a sensitivity analysis of the effect of O_3_ using the available data, including O_3_ in the model only marginally modified the associations with other pollutants or indicators (data not shown).

**Table 2:** Associations between daily emergency hospital visits for cardiorespiratory causes and one day of exposure to different sources of high PM_10_ values at lag 0–7. All models are adjusted for time trend, annual variation, climate and influenza season.

Indicator of PM_10_ Source	Percent Change in Emergency Hospital Visits *		Model
	% (95% CI)	*p*	*R^2^*
*All sources*	−0.7 (−2.6, 1.4)	0.519	0.23
-adjusted ** for PM_10_	−2.4 (−5.6, 0.8)	0.144	0.22
-adjusted for NO_2_ and H_2_S	0.2 (−2.2, 2.5)	0.892	0.24
-adjusted for NO_2_ H_2_S and PM_10_	−2.8 (−6.6, 1.2)	0.162	0.25
*Volcanic ash*	4.8 (0.6, 9.2)	0.024	0.23
-adjusted for PM_10_	6.1 (0.0, 12.7)	0.052	0.22
-adjusted for NO_2_ and H_2_S	6.2 (2.6, 12.5)	0.041	0.25
-adjusted for NO_2_ H_2_S and PM_10_	7.3 (−0.4, 15.5)	0.064	0.25
*Dust storm*	5.1 (0.5, 9.9)	0.028	0.23
-adjusted for PM_10_	5.8 (0.9, 19.9)	0.019	0.22
-adjusted for NO_2_ and H_2_S	2.1 (−3.3, 8.0)	0.444	0.25
-adjusted for NO_2_ H_2_S and PM_10_	1.2 (−4.6, 7.4)	0.692	0.25
*Other sources*	−3.3 (−5.6, −1.0)	0.007	0.23
-adjusted for PM_10_	−4.4 (−7.1, −1.7)	0.002	0.22
-adjusted for NO_2_ and H_2_S	−1.2 (−3.8, 1.5)	0.375	0.25
-adjusted for NO_2_ H_2_S and PM_10_	−3.6 (−6.9, −0.2)	0.036	0.25

* Estimated effects of 1 day with exposure during the lag period (0–7 days). ** n = 1941 for the unadjusted model, n = 1748 for the model adjusted for PM_10_, n = 1464 for the model adjusted for NO_2_ and H_2_S, n = 1403 for the model adjusted for three pollutants.

### 3.2. Discussion

Emergency hospital visits of adults for cardiorespiratory causes seemed to increase on days with high PM_10_ levels due to volcanic ash. The same was found for dust storms, though this ceased to be significant after adjusting for exposure to other pollutants. 

We cannot rule out that the observed association with volcanic ash is due to the extremely high levels of PM_10_ during some of these days. However, the PM_10_ levels were also very high on some of the dust storm days. For high PM_10_ due to other sources than volcanic ash or dust storms, we observed a small negative association with the outcome which was not statistically significant when adjusting for gaseous pollutants.

**Figure 2:**
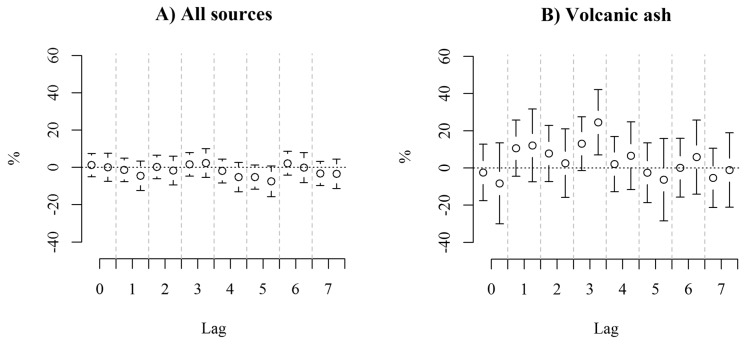

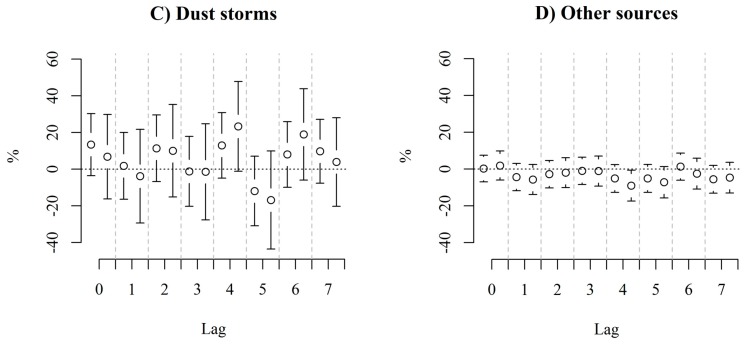
Associations between adult emergency hospital visits and indicators for PM_10_ over air quality guideline value due to (**A**) any source, (**B**) volcanic ash, (**C**) dust storms, (**D**) other sources. The effect estimates are shown unadjusted (left) and adjusted for PM_10_, NO_2_ and H_2_S (right).

Our finding that there were no associations between emergency hospital visits and dust storms are in contrast with those found for Saharan dust on respiratory disease admissions of 14.6% (95% CI 5.3 to 24.7%) per interquartile change in 0–5 day [37] but as our outcome is a conglomerate of cardiac, respiratory, and stroke events, the effect sizes are not easily comparable to other studies. Moreover, dust storms in Iceland might differ from dust storms of Saharan origin with respect to toxicity. Studies of mortality and exposure to dust storms in other settings found larger effects at shorter lag times than the current study [21,24,39]. In our study, lag 0–7 had the highest predictive power (supplementary table), but the effect estimates peaked at lag 3 for volcanic ash days, and at lag 4 for dust storm days (Figure 2). Possible mechanisms which could result in increased emergency hospital visits in exposed humans have been identified *in vitro*. Ash from Eyjafjallajökull has been found to compromise the function of rat alveolar macrophages, rendering the tissue more prone to infections [36], and with inflammatory responses at lag 2 to lag 5 [40].

#### 3.2.1. Strengths and Limitations

The most important strength of the study is the quality of the outcome data; emergency hospital visits are registered at a central hospital, which serves the entire region and the whole population which would reduce bias. The National Icelandic Registers are of high quality and the data thus consist of emergency hospital visits for the whole population of the Reykjavík capital area. All individuals in the study have been treated at the same center which increases the likelihood that the diagnoses have been attributed in a similar fashion during the whole period. The novel approach to separate ambient PM_10_ into different sources using meteorological methods as well as observations and back-tracking and the focus on long-transported volcanic ash are other strengths of the present study. 

#### 3.2.2. Statistical Power

To start, the results of the present study are somewhat inconclusive, perhaps given a low inherent statistical power to detect source specific associations due to few volcanic ash days and dust storm days. The results should thus be interpreted with caution. Another limitation is that most of the dust storm days occur before the Eyjafjallajökull volcanic eruption, and all of the volcanic ash days occur after, which might mean that the differences observed between volcanic ash days and dust storm days might be due to temporal variation, even though the statistical models were carefully adjusted for time trends. Stratifying the data into the before and after the Eyjafjallajökull volcanic eruption results in a lower inherent statistical power compared to analyzing the whole time period, which is why we refrained from doing that. PM_10_ levels during some volcanic ash days were extremely high at more than 500 µg/m^3^, but adjusting for PM_10_ did not attenuate the estimate of the volcanic ash days, so the effect of the ash is likely the cause of the observed association, rather than a high PM_10_ levels. Furthermore, PM_10_ levels during dust storm days were also occasionally extremely high.

#### 3.2.3. Missing Pollution Data

Another limitation is the quality of exposure data. All pollution data were collected at a roadside measuring station which is subject to un-proportionately high variation from traffic related air pollution, and our results may thus be subject to bias towards the null. Data from an urban background station would have been preferable, but that station had less complete data. Moreover, data on O_3_, a significant predictor of emergency hospital admissions in a previous study [26], were not available from 2010 and later and were thus excluded from the analysis altogether. In a sensitivity analysis of the effect of O_3_ using the available data, including O_3_ in the model only marginally modified the associations with other pollutants or indicators. For high PM_10_ due to other sources than volcanic ash or dust storm, we observed a small negative association with the outcome which persisted in both single-and multi-pollutant models. NO_2_ was substantially higher than average on days where the source for high PM_10_ was other sources than volcanic ash and dust storms, and the temperature was lower than average, why a potential explanation for the negative association might be residual confounding from lower levels of ozone than average in the latter time period. Data for PM_2.5_ were not available, but could have strengthened the source assignment of the high PM_10_ events. In the analysis of the source-specific pollution, we compare days with high levels due to certain sources with days where PM_10_ pollution was below the air quality guideline value. This could be problematic, but we attempted to accommodate this by presenting both adjusted and unadjusted results as well as estimates for high levels of PM_10_ from all sources, which was not associated with increased emergency hospital visits.

#### 3.2.4. Interpretation

The observed associations between emergency hospital admissions for cardiorespiratory emergency hospital visits following days with particle pollution due to volcanic ash are supported by findings of increased respiratory morbidity and hospital visits in other studies [18,19].

The discrepancies between adjusted and non-adjusted estimates were noticeable, especially for the estimates associated with volcanic ash and dust storms. High PM levels in Iceland’s capital occur especially during two very different meteorological conditions; either strong winds resuspended particle matter from the ground or blows in as dust storms from the eroded areas of south Iceland, or a lack of wind, stagnation, causes a build-up of traffic exhaust and particles—during which the levels of gaseous pollutants tend to be higher. The effect estimates of volcanic ash and dust storm days are both sensitive to adjusting for other pollutants (the associations decrease for dust storm, and increases for volcanic ash). In Table 1, we find that H_2_S is noticeably lower during dust storm days, which could account for the difference between adjusted and unadjusted estimates for dust storm days. 

The lack of associations with PM_10_ from other sources than volcanic ash or dust storms could be related to exposure misclassification. The measured PM_10_ value likely represents population exposure to PM_10_ better during conditions when exposure is distributed more evenly across the study area, such as during an ash or dust storm. Local resuspension and traffic pollution levels off quickly as distance from the source increases [31] and the PM_10_ value measured at the roadside will not be as representative for population exposure. 

All mineral particles in Iceland are in principle volcanic, thus, the particles responsible for dust storms contain older, weathered volcanic particles. A recent report found that the composition of PM_10_ in Reykjavík changed considerably from 2003 to 2013. In addition to the fresh volcanic ash the amount of brake matter and soot had increased from 2% and 7% in 2003 to 14% and 30% in 2013. The proportion of soil and asphalt had decreased from 25% and 55% in 2003 to 18% and 17% in 2013 respectively [41]. As both the sampling times for that study lay outside the study period of the current study, one can only speculate as to the impact of this change in PM_10_ composition on our results. 

Also, an analysis elemental composition of PM_10_ from the sampling filters could be considered. However, for time-series study using a long series of data, it would be unpractical, but could perhaps be used in combination with a model to refine the source assignment. Future studies are planned to study the transformation of volcanic ash as it weathers into other natural dust.

## 4. Conclusions 

In this epidemiological study on volcanic ash and other sources of particulate air pollution, we found some indications that high levels of PM_10_ due to volcanic ash increased the number of emergency hospital visits for cardiorespiratory causes in adults in Reykjavík, Iceland. However, the effect estimate was only borderline statistically significant and the results should be interpreted with caution.

## Supplementary Files

Supplementary File 1
