# Supplementary material for: Emergency Hospital Visits in Association with Volcanic Ash, Dust Storms and Other Sources of Ambient Particles: A Time-Series Study in Reykjavík, Iceland
Source: Int J Environ Res Public Health. 2015 Apr 13;12(4):4047–59. doi: 10.3390/ijerph120404047 (PMC4410232; doi:10.3390/ijerph120404047)
Supplement: Supplementary File 1 [file ijerph-12-04047-s001.pdf]

Supplementary Information

Emergency Hospital Visits in Association with Volcanic Ash, Dust Storms and Other Sources of Ambient Particles: A Time-Series Study in Reykjavík, Iceland

Table S1. Associations between daily emergency hospital visits for cardiorespiratory causes for one day of exposure to different sources of high PM_{10} values at different lag intervals 0–7. All models are adjusted for time trend, annual variation, climate and influenza season, but not other pollutants.

PM_{10} Source	Percent Change in Emergency Hospital Visits *		Model
	%	*p*	R^2
All sources			
-Lag0	1.9	0.54	0.22
-Lag02	0.9	0.61	0.22
-Lag05	−0.3	0.78	0.21
-Lag07	−0.6	0.57	0.21
Volcanic ash			
-Lag0	4.9	0.58	0.22
-Lag02	8.4	0.04	0.22
-Lag05	7.2	0.01	0.22
-Lag07	5.1	0.02	0.22
Natural dust			
-Lag0	13.6	0.11	0.23
-Lag02	8.6	0.04	0.22
-Lag05	6.1	0.05	0.21
-Lag07	5.7	0.02	0.21
Other sources			
-Lag0	−0.2	0.95	0.22
-Lag02	−2.1	028	0.22
-Lag05	−3.3	0.02	0.22
-Lag07	−3.4	0.01	0.21

© 2015 by the authors; licensee MDPI, Basel, Switzerland. This article is an open access article distributed under the terms and conditions of the Creative Commons Attribution license (http://creativecommons.org/licenses/by/4.0/).
